# Maternal exposure to airborne polychlorinated biphenyls (PCBs) and risk of adverse birth outcomes

**DOI:** 10.1007/s10654-021-00793-x

**Published:** 2021-08-22

**Authors:** Ane Bungum Kofoed, Laura Deen, Karin Sørig Hougaard, Kajsa Ugelvig Petersen, Harald William Meyer, Ellen Bøtker Pedersen, Niels Erik Ebbehøj, Berit Lilienthal Heitmann, Jens Peter Bonde, Sandra Søgaard Tøttenborg

**Affiliations:** 1 Department of Occupational and Environmental Medicine, Bispebjerg and Frederiksberg Hospital, Bispebjerg Bakke 23, Building 20F, 2400 Copenhagen, Denmark; 2 National Research Centre for the Working Environment, Copenhagen, Denmark; 3 Department of Public Health, Faculty of Health, University of Copenhagen, Copenhagen, Denmark; 4 Research Unit for Dietary Studies, The Parker Institute, Bispebjerg and Frederiksberg Hospital, Copenhagen, Denmark; 5 Section for General Practice, Department of Public Health, Faculty of Health, University of Copenhagen, Copenhagen, Denmark

**Keywords:** PCB, Lower chlorinated PCBs, Airborne PCB, Maternal exposure, Birth outcomes, Cryptorchidism

## Abstract

Human health effects of airborne lower-chlorinated polychlorinated biphenyls (LC-PCBs) are largely unexplored. Since PCBs may cross the placenta, maternal exposure could potentially have negative consequences for fetal development. We aimed to determine if exposure to airborne PCB during pregnancy was associated with adverse birth outcomes. In this cohort study, exposed women had lived in PCB contaminated apartments at least one year during the 3.6 years before conception or the entire first trimester of pregnancy. The women and their children were followed for birth outcomes in Danish health registers. Logistic regression was performed to estimate odds ratios (OR) for changes in secondary sex ratio, preterm birth, major congenital malformations, cryptorchidism, and being born small for gestational age. We performed linear regression to estimate difference in birth weight among children of exposed and unexposed mothers. All models were adjusted for maternal age, educational level, ethnicity, and calendar time. We identified 885 exposed pregnancies and 3327 unexposed pregnancies. Relative to unexposed women, exposed women had OR 0.97 (95% CI 0.82, 1.15) for secondary sex ratio, OR 1.13 (95% CI 0.76, 1.67) for preterm birth, OR 1.28 (95% CI 0.81, 2.01) for having a child with major malformations, OR 1.73 (95% CI 1.01, 2.95) for cryptorchidism and OR 1.23 (95% CI 0.88, 1.72) for giving birth to a child born small for gestational age. The difference in birth weight for children of exposed compared to unexposed women was − 32 g (95% CI—79, 14). We observed an increased risk of cryptorchidism among boys after maternal airborne LC-PCB exposure, but due to the proxy measure of exposure, inability to perform dose–response analyses, and the lack of comparable literature, larger cohort studies with direct measures of exposure are needed to investigate the safety of airborne LC-PCB exposure during pregnancy

## Background

PCBs are some of the most widespread persistent organic pollutants in the environment due to their extensive use in electrical equipment and building materials from late 1920s to the 1970s and their continuous release from waste and building materials from that period [1–3]. After decades of use, PCBs were banned after concern about bioaccumulation and cancer risks. Since their ban, much attention has been paid to the accumulation of higher chlorinated PCBs (HC-PCBs) in the food chain, which is thought to constitute the most important source of human exposure. Studies have demonstrated conflicting yet concerning results in regards to health effects of these PCBs [4–9]. It was however recently discovered that residents of PCB contaminated apartments have considerably higher blood levels of lower chlorinated PCBs (LC-PCBs) compared to residents of uncontaminated apartments [10, 11], and as much as 63% of the total PCB body burden in exposed adults and 36% in exposed toddlers may be from inhaled LC-PCBs [12].

Yet, human health effects of LC-PCBs remain largely unexplored [13, 14]. HC-PCBs and LC-PCBs have inherently different toxicological profiles [15]. Studies of HC-PCBs can therefore not be directly used as basis for e.g. action levels for LC-PCBs, even if this is presently the case for private housing and workplaces in Denmark [14]. The few studies that exist on the toxicity of LC-PCBs are associated with cancer, metabolic and reproductive disorders [15–18] and indicate that they have estrogenic and anti-androgenic properties [15, 19]. LC-PCBs can furthermore cross the placenta [20], and at a higher rate than HC-PCBs [21] and might therefore have potential to negatively affect fetal development [22, 23].

To date, no previous study has investigated if living in PCB contaminated buildings is safe for the pregnant woman and her child. If LC-PCBs can disrupt hormonal balance, plausible consequences could be changes in secondary sex ratio, increased risk of preterm birth, major malformations, and changes in fetal growth [24]. We therefor aimed to determine, if pre-pregnancy—and first trimester exposure to LC-PCBs was associated with adverse birth outcomes. We used nested data from the Health Effects of PCBs in Indoor Air (HESPAIR) cohort, which included 3307 women with 4212 pregnancies carried in estates partly contaminated with PCBs.

## Methods

### Study population

The current study was nested in the HESPAIR cohort, specifically designed to study health effects after exposure to PCB in indoor air. The cohort was established in 2019 by the Department of Occupational and Environmental Medicine (Bispebjerg Hospital, Denmark). HESPAIR consists of 52 212 former or current residents of the building estates Farum Midtpunkt and Brøndby Strand Parkerne during the period 1970–2018. The estates were erected during 1969–1974 and include both PCB contaminated apartments and uncontaminated apartments. This is because PCB containing caulks and sealants were used during the first stages of construction, while other building materials were used in later stages [10]. Measurements of PCB concentrations in indoor air in contaminated and uncontaminated estates have been conducted and revealed large differences and differences were dominated by LC-PCBs [3, 25]. Plasma levels of PCBs with 3–4 chlorine atoms were over 50 times higher among 134 exposed compared to 139 unexposed residents [10, 11]. Further, the median level of serum PCB-28 (LC-PCB) were 70 times higher among 71 exposed residents compared to 23 unexposed residents.

### Inclusion and exposure

In the present study, we included all women from the HESPAIR cohort who had lived in the estates up to or during their pregnancy by linking unique civil registration numbers from the HESPAIR cohort to the Danish Medical Birth Registry (MBR) [26]. We identified 10 226 women who had had one or more pregnancies resulting in live born singletons registered *after* their first relocation to the estates. The MBR holds data on health of pregnant women and their offspring, and all live and still births in Denmark. The register was established in 1973 and has been updated continuously. It underwent a major revision in 1997, where the register became electronic, and a list of new variables was added [26].

**Fig. 1:**
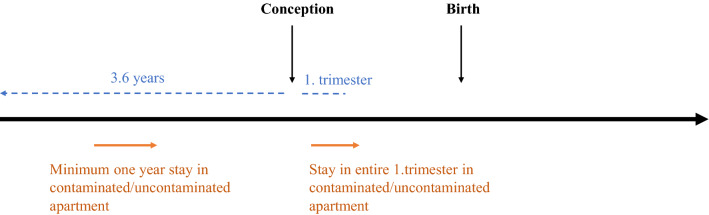
Demonstrating exposure/unexposure criteria of minimum one year stay in a contaminated/uncontaminated apartment during the 3.6-year period leading up to conception or during the entire first trimester. Additionally, unexposed could not have lived in a contaminated apartment in the two building estates previously or during the period of interest

The Danish Central Person register (CPR) was used to extract information on dates and addresses of relocations. This enabled calculation of timing and duration of living in the estates, which served as a proxy for PCB exposure. Women were defined as exposed if they had lived in a contaminated apartment for at least one year during the 3.6-year period leading up to conception or during the entire first trimester (Fig. 1). Conception was defined as the child’s birth date minus gestational age, or child’s birth date minus 38 weeks (266 days) if gestational age was unavailable, e.g. before 1978. The same criteria were applied for women to be considered unexposed, but in an uncontaminated apartment, with the additional criterium that they could not have lived in a contaminated apartment in the two building estates previously or during the period of interest for this study.

The one year exposure criterium was based on a reanalysis of the data on plasma concentration of PCB-28 among 134 exposed and 139 non-exposed residents, obtained by Meyer et al. [10] that showed significantly higher plasma concentrations of LC-PCBs among residents after one year of living in contaminated apartments. Unfortunately, plasma samples were not available for this study’s nested population. The time window of 3.6 years was constructed from the half-life of PCB-28. This varies according to different studies, but one study found the half-life to be 4.6 years [27], one study reported 5.5 [28], and other studies reported shorter half-lives (1.4 and 3 years) [29, 30]. The time window of 3.6 years allowed the mother to live in a contaminated apartment and relocate to an uncontaminated apartment up to 2.6 years prior to conception, while the woman and her future child would still be exposed to over half of the accumulated LC-PCB. The exposure criterium regarding the first trimester was included to reflect an ongoing exposure during establishment of the pregnancy and organogenesis, which is a very sensitive stage of pregnancy, among others for hormonal disruption and development of the reproductive organs [23].

### Birth outcomes

Birth outcomes were retrieved from birth records in the MBR available since 1973 and hospital contacts in the Danish National Patient Register (DNPR) available since 1977. The DNPR holds information on diagnoses and treatments for all patients admitted to any public or private hospital and was used to retrieve data on congenital malformations [31].

The child’s sex was retrieved from birth records of live births, to examine secondary sex ratio. Secondary sex ratio was expressed as the chance of having a live born son. This is of interest since hormonal disruption could decrease the prevalence of boys [32]. Information on preterm birth, defined as being born before 37 completed weeks of gestation [33], was identified using gestational age registered and available in birth records. Major congenital malformations were defined after guidelines by European Surveillance of Congenital Anomalies (EUROCAT) [34]. Major malformations were included if diagnosed within the first year of life and registered with a diagnose code in the DNPR (from 1977) and the MBR (from 1997). Only the first diagnose on any type of major malformation was included. Minor malformations, also defined as by EUROCAT [35], were excluded. Cryptorchidism is defined as a minor malformation, but is of specific interest as an indicator of hormonal disruption of testicular development [36]. It was therefore included in a separate analysis. Cryptorchidism was identified in the MBR and DNPR using the ICD-8 codes: 75210, 75211, 75219, and ICD-10 codes: Q530, Q531, Q531A, Q532, Q532A or Q539, respectively, or registration with Surgery and Treatment Classification of the National Board of Health: 55,640 and Nordic Classification of Surgical Procedures: KFH00, KFH01 and KFH10, respectively. We included first diagnosis of cryptorchidism from birth until 2018, since this type of malformation can be diagnosed several years after birth [37, 38]. Birth weight for gestational age and birth weight on a continuous scale was investigated in two separate analyses. Small for gestational age (SGA) was determined using birth weight for gestational age, where SGA is defined as birth weight below the 10th percentile for each gestational week, stratified by sex [39]. SGA was calculated by the commonly used Kramer method [40] with birth weight, gestational age in weeks, and sex retrieved from birth records.

### Covariates

**Fig. 2:**
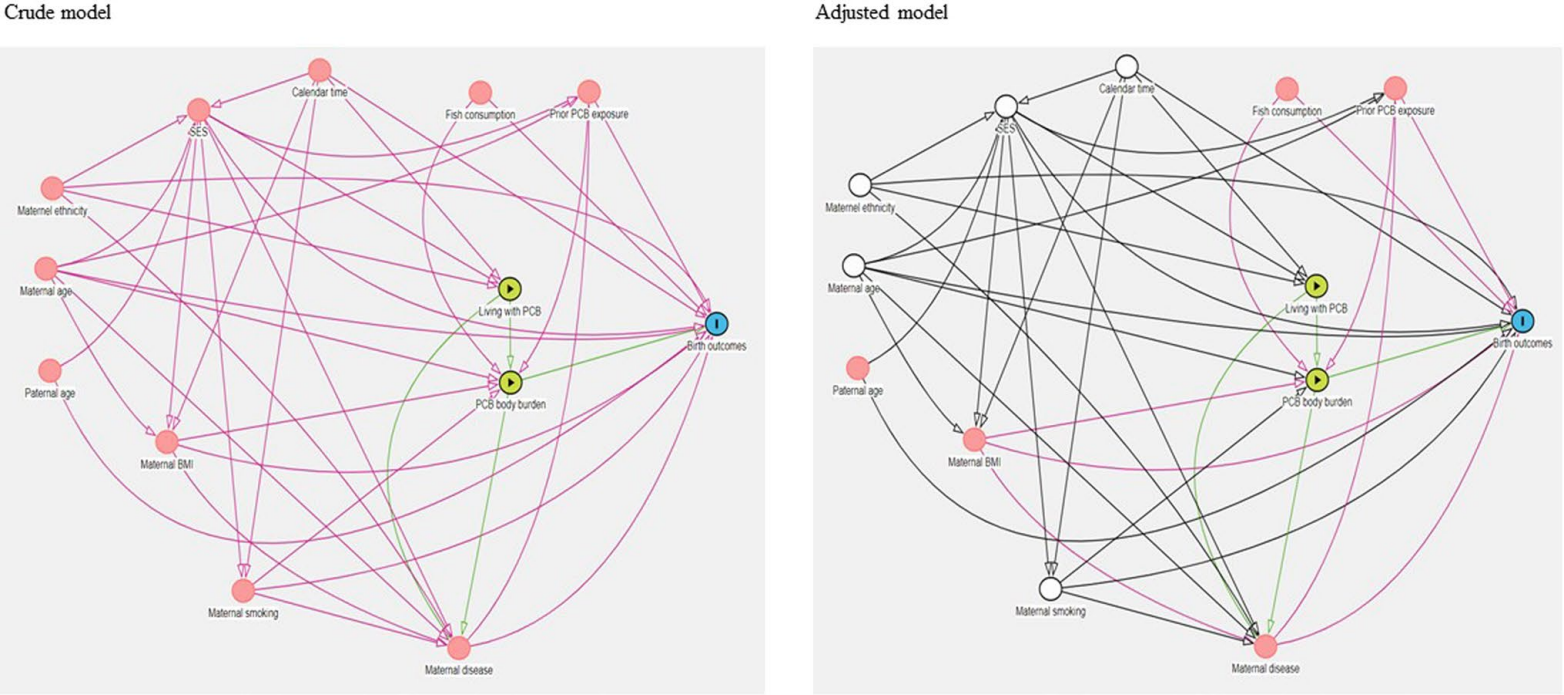
Show confounders identified a priori in crude (left) and adjusted (right) model. After adjusting for confounders indicated by white circles (in adjusted model), the purple arrows show remaining biasing paths from maternal BMI to exposure and outcome

Potential confounders were identified a priori using directed acyclic graphs [41], see Fig. 2. We distinguished between two aspects of exposure since we assumed PCB exposure status (living in a contaminated/uncontaminated resident) was a proxy of PCB body burden, and confounders were differently associated with these two aspects. We included maternal age as a continuous variable was obtained from the MBR. Maternal education, categorized as low, middle, and high, and ethnicity + , categorized as coming from the Nordic countries or not, were obtained from the CPR register. We also adjusted for calendar time in decades. Smoking information was available from the MBR since 1991.

### Statistical analysis

In analyses regarding secondary sex ratio, SGA, preterm birth, major congenital malformations, and cryptorchidism we performed logistic regression with adjustment for maternal age, educational level, ethnicity, and calendar time. We restricted the analyses of preterm birth and birth weight to children born from 1982, since before 1982 we found many missing values on gestational age and birth weight was coded in large weight intervals (e.g. 250 g intervals), as reported previously [42]. In addition to the overall analysis on cryptorchidism, we restricted one analysis to children born full term (week 37–42), since the risk of cryptorchidism is highly dependent on gestational age. Risk estimates were expressed as odds ratios (ORs) with 95% confidence intervals (95% CI). Correlation between siblings were accounted for in all analyses by applying robust standard errors when calculating confidence intervals. Birth weight was normally distributed, and we therefore performed multiple linear regression, expressing difference in birth weight as β-coefficient with 95% CI. In the multiple linear regression, we also checked for interaction between exposure and sex. In addition to the overall analysis on birth weight, we restricted one analysis to children born full term (week 37–42). To take potential confounding by maternal smoking up to pregnancy into account, we repeated all analyses with adjustment for smoking among women who had given birth between 1991 and 2018, because smoking information (no/yes) was registered only during this period. For all outcomes, we also checked for interaction between LC-PCB exposure and smoking. We furthermore repeated all analyses while restricting to nulliparous women, since nulliparous women represent a more homogenous group where previous experience with pregnancy and birth does not influence the desire and motivation for having more children. Also, nulliparous women have previously been reported to have higher risks for negative birth outcomes [43]. All statistical tests were performed using Stata software (version 16.0, Stata Corp, College Station, TX).

## Results

**Fig. 3:**
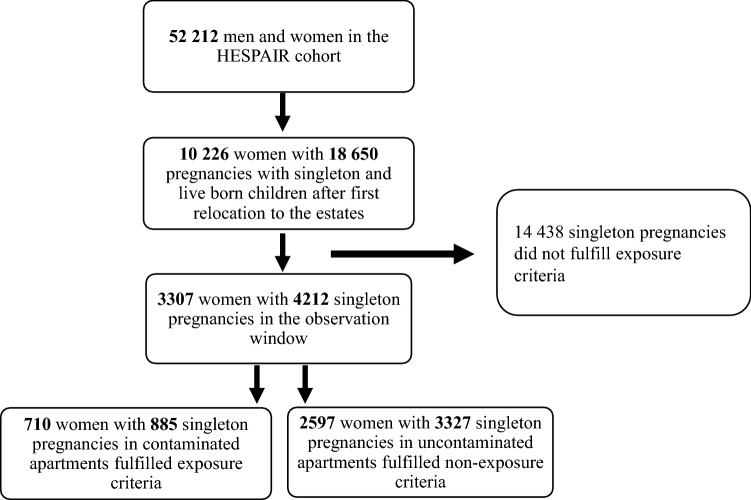
Flow chart of identification process of exposed and unexposed residents

In the HESPAIR cohort we identified 10 226 women who had 18 650 pregnancies with live born singletons registered *after* their first relocation to the estates. Of these, only 3307 women and their 4212 pregnancies fulfilled the criteria of being exposed or unexposed during the defined observation window, of which 885 (21%) pregnancies were exposed (see Fig. 3).

### Maternal characteristics

Mean age of mothers were similar among exposed and unexposed (Table 1). We found a small difference in ethnicity, since more exposed compared to unexposed were from Nordic countries (68% versus 64%). Fewer exposed were represented in the lowest and highest educational group compared to unexposed. However, among the exposed, a higher percentage had missing data on educational level compared to unexposed (29% versus 21%). The first decade, 1970–1979, represented more pregnancies by exposed women compared to unexposed (16% versus 11%), and the last decade 2010–2018 represented more pregnancies by unexposed women compared to exposed (14% versus 10%). In data regarding maternal smoking up to pregnancy available since 1991, we found no difference in distribution.

**Table 1:** Baseline characteristics for 3307 mothers at the time of 4212 pregnancies

	Unexposed (reference)	Exposed
*n* pregnancies (*%*)	3327 (79)	885 (21)
Maternal age (SD)	27.9 (5.4)	27.5 (5.4)
Maternal ethncity		
Nordic contries %	64	68
Non-nordic contries %	36	32
Missing %	0	0
Maternal level of education		
Low %	35	30
Middle %	27	26
High %	17	15
Missing %	21	29
Calendar time of conception		
1970–1979%	11	16
1980–1989%	27	29
1990–1999%	27	26
2000–2009%	21	19
2010–2018%	14	10
Maternal smoking up to pregnancy^a^		
No %	69	68
Yes %	23	22
Missing %	8	10

*SD* standard deviation

^a^Available since 1991 (unexposed *n* = *2028 and* exposed *n* = 476)

### Secondary sex ratio

We found no difference in secondary sex ratio in neither crude nor adjusted analyses (OR 0.97 with 95% CI 0.82, 1.15) (Table 2). Smoking did not interact with LC-PCB exposure, and adjustment for smoking and restriction to first born children did not change results.

**Table 2:** Odds ratios (OR) with 95% confidence intervals (CI) of adverse birth outcomes among 885 pregnancies exposed to LC-PCB relative to 3327 unexposed

	n cases (%)	Crude model	Adjusted model^a^	Restricted to children born 1991–2018^b^	Restricted to first born children^a^
		OR (95% CI)	OR (95% CI)	OR (95% CI)	OR (95% CI)
Secondary sex ratio					
Unexposed	1689 (51)	1 (ref)	1 (ref)	1 (ref)	1 (ref)
Exposed	442 (50)	0.97 (0.84, 1.12)	0.97 (0.82, 1.15)	0.96 (0.77, 1.19)	0.99 (0.80, 1.23)
Preterm birth					
Unexposed	146 (5)	1 (ref)	1 (ref)	1 (ref)	1 (ref)
Exposed	37 (5)	1.02 (0.70, 1.49)	1.13 (0.76, 1.67)	1.20 (0.75, 1.92)	1.08 (0.69, 1.71)
Major malformation					
Unexposed	114 (3)	1 (ref)	1 (ref)	1 (ref)	1 (ref)
Exposed	30 (4)	1.01 (0.66, 1.53)	1.28 (0.81, 2.01)	1.13 (0.66, 1.96)	1.37 (0.80, 2.35)

*LC-PCB* lower chlorinated polychlorinated biphenyls, *ref* reference, *OR* odds ratio, *CI* confidence interval

^a^Adjusted for maternal age, highest level of education, ethnicity, and calendar time

^b^Adjusted for maternal age, highest level of education, ethnicity, calendar time, and smoking

### Preterm birth

We found no difference in risk of preterm birth (before 37 completed weeks of gestation) (OR 1.13 with 95% CI 0.76, 1.67) (Table 2). Smoking did not interact with LC-PCB, but adjustment for smoking elevated the risk estimate to 25% (OR 1.25 with 95% CI 0.73, 2.14) but with a wider CI. Restriction to first born children was in line with the result from the main analysis.

### Major malformations

In adjusted analyses we observed an indicated 28% higher risk of major malformations (OR 1.28 with 95% CI 0.81, 2.01) (Table 2). Smoking did not interact with LC-PCB, and risk attenuated when adjusting for smoking (OR 1.13 with 95% CI 0.66, 1.96). The results were robust to restriction to first born children.

### Cryptorchidism

In adjusted analyses, we found that women exposed to LC-PCBs had a 73% higher risk of giving birth to sons with cryptorchidism but with a wide confidence interval (OR 1.73 with 95% CI 1.01, 2.95) (Table 3). No interaction between LC-PCB exposure and smoking was seen, and the higher risk remained after adjustment for smoking and restriction to first born children in the sensitivity analyses. Similarly, when looking at risk of cryptorchidism among boys born full term, exposed pregnancies had a 75% higher risk compared to unexposed but still with a wide confidence interval (OR 1.75 with 95% CI 1.01, 3.04).

**Table 3:** Odds ratios (OR) with 95% confidence intervals (CI) of cryptorchidism among boys of 442 pregnancies exposed to LC-PCB relative to 1689 unexposed

	*n* cases (%)	Crude model	Adjusted model^a^	Restricted to children born 1991–2018^b^	Restricted to first born children^a^	Restricted to children born full term^c^
		OR (95% CI)	OR (95% CI)	OR (95% CI)	OR (95% CI)	OR (95% CI)
Cryptorchidism						
Unexposed	66 (4)	1 (ref)	1 (ref)	1 (ref)	1 (ref)	1 (ref)
Exposed	25 (6)	1.47 (0.92, 2.36)	1.73 (1.01, 2.95)	1.77 (0.96, 3.27)	2.18 (1.13, 4.21)	1.75 (1.01, 3.04)

*LC-PCB* lower chlorinated polychlorinated biphenyls, *ref* reference, *OR* odds ratio, *CI* confidence interval

^a^Adjusted for maternal age, highest level of education, ethnicity, and calendar time

^b^Adjusted for maternal age, highest level of education, ethnicity, calendar time, and smoking

^c^Adjusted for maternal age, highest level of education, ethnicity, and calendar time

### Birth weight

In adjusted analyses, LC-PCB exposure indicated a 23% increased risk giving birth to a SGA child (OR 1.23 with 95% CI 0.88, 1.72) (Table 4). No interaction between exposure and smoking was found. Adjustment for smoking gave essentially similar results (OR of 1.34 (95% CI 0.88, 2.03)). However, when examining SGA among first born children, the risk was attenuated.

**Table 4:** Odds ratios (OR) with 95% confidence intervals (CI) of having a child born small for gestational age among 686 pregnancies exposed to LC-PCB relative to 2794 unexposed

	*n* cases (%)	Crude model	Adjusted model^a^	Restricted to children born 1991–2018^b^	Restricted to first born children^a^
		OR (95% CI)	OR (95% CI)	OR (95% CI)	OR (95% CI)
Small for gestational age					
Unexposed	239 (9)	1 (ref)	1 (ref)	1 (ref)	1 (ref)
Exposed	68 (10)	1.18 (0.87, 1.60)	1.23 (0.88, 1.72)	1.34 (0.88, 2.03)	1.04 (0.72, 1.51)

*LC-PCB* lower chlorinated polychlorinated biphenyls, *ref* reference, *OR* odds ratio, *CI* confidence interval

^a^Adjusted for maternal age, highest level of education, ethnicity, and calendar time

^b^Adjusted for maternal age, highest level of education, ethnicity, calendar time, and smoking

Adjusted analyses of birth weight on a continuous scale showed that the difference in birth weight was − 32 g (β-coefficient − 32 with 95% CI:—79, 14) among children of exposed compared to children of unexposed women (Table 5), and no difference in birth weight was observed across sex. When restricting the analyses to full term children (i.e. born week 37–42), the same tendency was observed (Table 5). The tendency was also observed when adjusting for smoking and when restricting analysis to first born children. No interaction between exposure and smoking was observed.

**Table 5:** Linear regression of birth weight among 686 pregnancies exposed to LC-PCB relative to 2794 unexposed

	Mean g (SD)	Crude	Adjusted^b^	Adjusted^c^	Restricted to children born 1991–2018^d^	Restricted to first born children^c^
		β-coefficient (95% CI)	β-coefficient (95% CI)	β-coefficient (95% CI)	β-coefficient (95% CI)	β-coefficient (95% CI)
All children						
Unexposed	3457 (555)	(ref)	(ref)	(ref)	(ref)	(ref)
Exposed	3460 (527)	3 (− 46, 52)	− 30 (− 74, 15)	− 32 (− 79, 14)	− 29 (− 86, 28)	1 (− 47, 50)
Full term children^a^						
Unexposed	3509 (499)	(ref)	(ref)	(ref)	(ref)	(ref)
Exposed	3506 (480)	− 3 (− 50, 44)	− 28 (− 74, 17)	− 32 (− 80, 16)	− 30 (− 89, 28)	− 25 (− 88, 37)

*LC-PCB* lower chlorinated polychlorinated biphenyls, *g* grams, *SD* standard deviation, *ref* reference, *CI* confidence interval

^a^Unexposed *n* = 2642 and exposed *n* = 654

^b^Adjusted for gestational age

^c^Adjusted for gestational age, sex, maternal age, highest level of education, ethnicity, and calendar time

^d^Adjusted for gestational age, sex, maternal age, highest level of education, ethnicity, calendar time and smoking

## Discussion

### Main results

In this first study ever to investigate the relationship between pre-pregnancy and first trimester exposure to airborne LC-PCBs and adverse birth outcomes, we found increased risk of cryptorchidism in sons.

### Previous literature and mechanisms

As the historic focus has been on HC-PCB congeners, no previous evidence related to airborne LC-PCB exposure and birth outcomes is available. Evidence on HC-PCB and birth outcomes is conflicting, which may be due to the numerous differences across the previous studies, e.g. study design, population characteristics, measure of and differences in level of exposure [4, 6–9, 39, 44–48]. Currently, the risk assessment of airborne PCB exposure is based on the knowledge obtained on HC-PCBs. Due to differences in toxicological profiles between HC- and LC-PCBs [15], and the faster rates of placental transfer for LC-PCBs [21], potential health effects of LC-PCBs need to be assessed separately.

Very few studies have specifically examined the toxicological mechanisms of LC-PCBs. Based on their mechanisms, the overall group of the 209 PCB congeners can be divided into dioxin like PCBs (DL-PCBs), and non-dioxin like PCBs (NDL-PCBs) which includes most HC-PCBs and LC-PCBs. Like dioxins, the 12 existing DL-PCBs (PCB 77, 81, 105, 114, 118, 123, 126, 156, 157, 167, 169, and 189) act through the Aryl Hydrocarbon Receptor (AhR). NDL-PCBs probably act on multiple other receptors and pathways, for instance the constitutive androstane receptor (CAR) and the pregnane X receptor (PXR) [49]. The effects of interaction with CAR and PXR can be modulated gene expression linked to estrogenic, androgenic and thyroid receptors, and altered hormonal homeostasis [19]. During metabolism of NDL-PCBs, hydroxylated metabolites (OH-NDL-PCBs) may be formed [50], and these can also act on the estrogen and the androgenic receptors [51]. The existing evidence points to different toxicological mechanism of LC—and HC-PCBs. One in vitro study examined the difference between LC and HC-PCBs, and reported LC-PCBs to have estrogenic effects, while HC-PCBs mainly had anti-estrogenic effects [15]. Another in vitro study, found LC-PCBs to have affinity for CAR, PXR, be highly anti-androgenic and estrogenic, and LC-PCB metabolites were found to have greater estrogenic effects than their parent congener [19]. These in vitro studies have been supported by one cross-sectional study on PCB concentration in umbilical cord sera and adverse birth outcomes. This study observed different effects according to the degree of chlorination, and found LC-PCBs to be associated with lower levels of luteinizing hormone and testosterone, lower gestational age and smaller head circumference [52].

We observed an increased risk of cryptorchidism for exposed pregnancies, but with a wide 95% confidence interval, which implies uncertainty of the risk estimate and could be due to chance but could also be due few cases in a population with a moderate size. However, a plausible mechanism could be the hypothesized estrogenic and anti-androgenic activity of LC-PCBs during the ‘male programming window’ in the first trimester [53]. Previous cross-sectional and case–control studies on the relationship between HC-PCB and risk of cryptorchidism have demonstrated conflicting results [54–56]. No previous studies have examined the relationship between LC-PCBs and cryptorchidism, but one study found that maternal blood level of two LC-PCB congeners (PCB-28 and PCB-74) in the third trimester was associated with shorter anogenital distance (AGD) in newborn boys [18]. AGD is considered a marker of endocrine disruption and reproductive disease [57, 58]. Tang and colleagues found LC-PCBs to be associated with lower levels of luteinizing hormone and testosterone [52], which are crucial hormones in the process of testicular descent in fetal life [59].

We observed an indication of impaired organogenesis after airborne LC-PCB exposure, but again with a wide 95% confidence interval. However, as we only included major malformations diagnosed within the child’s first year, we could have missed some cases as the registration of diagnose codes in the DNPR started in 1977, and the estates—with contaminated apartments being built first—were available for living from the beginning of the 1970’ies. Nevertheless, when we studied children born 1991–2018, where registration of malformations and maternal smoking information were available for the whole period, we found an attenuated risk estimate. Teratogenic effects have been observed in ‘Yucheng’ children after their mothers accidently ingested polychlorinated dibenzofurans (PCDFs)—and PCB contaminated rice oil in 1978–1979 [60]. ‘Yucheng’ children were prenatally exposed to very high concentrations of PCB (but primarily HC-PCBs) and PCDFs. Similarly, other persistent organic pollutants (POPs) have been associated with certain congenital malformations [48, 61].

We found no increased risk of preterm birth, and the main analysis was supported by the analysis among first born children where we expected the mothers to represent a more homogenous group independent of previous experiences with pregnancy and birth,. This is in contrast to a previous cross-sectional study reporting an inverse association between LC-PCBs in umbilical cord blood and gestational age at birth [52].

We also found indications of affected gestational growth, but once again with wide 95% confidence intervals. If gestational growth is affected by LC-PCBs, it may be explained by a wide range of effects of PCBs on metabolism, e.g. alteration of insulin regulation [62], and thyroid, antiandrogenic and estrogenic effects [63, 64]. But again, when analysis was restricted to first born children, birth weight differences diminished, which weakens our hypothesis of effect on fetal growth. Studies on prenatal HC-PCB exposure and birth weight reports conflicting results [6, 8, 44, 46], and some studies found the effect to be dependent on maternal smoking and sex of the child [6, 9]. We did not observe interaction between exposure and smoking, nor exposure and sex of the child, as reported after HC-PCBs exposure [6].

### Problem magnitude and perspectives

Indoor LC-PCB contamination in private homes and public buildings is a global issue including countries like Germany and The United States [65–67]. Presently, 0.7–1.5% of all buildings in Denmark have indoor air levels exceeding the lower recommended action level, but the problem might be of larger scale since 37% of the total housing stock was constructed during the period when PCBs were allowed and commonly used (1950–1979) [68]. Studies examining schools and office buildings found concentrations exceeding the limit in many locations [66, 67, 69]. Since the action limit for PCB concentration in indoor air is based on knowledge on toxicity of HC-PCBs, this value may be inadequate, and the public health issue might be of much larger scale. Our results call for larger cohort studies investigating the existing limit value and the safety of staying in PCB contaminated estates and workplaces.

### Strengths and limitations

Our study has several strengths. The relationship was studied in a residence-based cohort, where people have unknowingly relocated themselves to contaminated and uncontaminated apartments, except for the first years of the cohort period where contaminated apartments were available first. Since contaminated apartments were built first, the chance of moving into a contaminated apartment were naturally higher during this period. Nevertheless, we expect residents from the estates to be similar in background exposure to PCBs, socioeconomic status, and health behavior, which previous studies on subsamples of the total HESPAIR cohort have demonstrated [10, 11]. We did however adjust for calendar time, as the quality of registration and sociodemographic characteristics of residents may have changed over the five decades of inclusion. Both maternal age at giving birth and smoking habits have changed over time and is related to birth outcomes. We therefore considered maternal age a confounder to be adjusted for (Fig. 2). Smoking could also be related to the susceptibility to PCB exposure, and due to the afore mentioned reason be related to PCB body burden (Fig. 2), and smoking is related to the risk of negative birth outcomes [50]. Therefore, smoking could interact with the association between exposure and outcome, but also confound the association. In our sensitivity analysis restricted to children born 1991–2018, where smoking information was available, no interaction was observed. Preferably, and as suggested in our directed acyclic graph (Fig. 2), body mass index (BMI) should also have been adjusted for, because mobilization of body fat during pregnancy can release accumulated PCBs to the bloodstream [18], but BMI was only available since 2004 and were missing for half of the residents. Nevertheless, we expect residual confounding regarding maternal body mass index (BMI), and maternal and paternal smoking to be minimal, as previous studies on subsamples of estate residents have demonstrated equal distribution of BMI and smoking among exposed and unexposed residents [10, 11]. We were not able to adjust for possible confounders such as paternal age and fish consumption, but we do believe these variables to be equally distributed among exposed and unexposed, in line with other variables, and residual confounding from these variables is expected to be minimal. Adjustment for maternal education, which can be the most appropriate measure of socioeconomic status for young adults who have yet to establish themselves occupationally [49], also minimized the chance of residual confounding as it is closely related to health behavior, including BMI and smoking. We did however have more missing data on educational level among exposed women which could be due to educational data being available since 1981, and more exposed compared to unexposed pregnancies were represented before this (1970–1980). In our sensitivity analysis restricted to children born 1991–2018, data on educational level was available for the full period, and the difference in missing data was reduced.

The registers made it possible to avoid self-report of outcomes, but also presented some issues. First, the DNPR, the only source of information on congenital malformations prior to 1997, did not cover the entire cohort period, as the register was established in 1977, and the estates were available since the beginning of the 1970’s. Further, registration methods, quality, and especially the registration completeness in the MBR changed over time [26]. For instance, the number of infants registered with congenital malformations have increased over time, and this is likely due to more systematic examination and improved registration [70]. We attempted to reduce the difference over time by adjusting for calendar time. Also, the imprecise registration of gestational age (e.g. full term/not full term and many missing values) and birth weight (e.g. intervals of 250 g) in the beginning of the MBR [42] caused us to restrict the analyses on birth weight to children born 1982–2018. Furthermore, data did not allow for a direct measure of exposure, e.g. of PCB levels in blood (PCB body burden), and we could therefore not isolate and discriminate effects of individual congeners or groups of congeners or investigate a dose–response relationship. Instead of a direct measure of PCB body burden, we used residential status as a proxy for total PCB exposure in the observational window of 3.6 years up to conception (based on the half-life of PCB-28), which is associated with several insecurities. Also, the lack of a direct measure of PCB body burden allows the risk of residual confounding from PCB exposure prior to the observational window, and we cannot reduce exposure to pre-pregnancy and first trimester only, as mothers may likely have lived in their respective apartments the whole pregnancy. Also, we do not have information about how residents of the estates likely have interacted with each other, and unexposed women may have spent time in contaminated apartments and vice versa for exposed. This could have caused non-differential misclassification and have diluted the association between exposure and birth outcomes.

Lastly, a common source of bias is the ‘live birth bias’ arising when studying prenatal exposure [71]. Only 60–70% of fertilized eggs will result in live births, and if the exposure is related to fetal survival and chance of live birth, the consequences of exposure (especially for major malformations) could be underestimated. But when adjusting for factors that could be related to fetal survival, e.g. smoking and maternal age, the risk of this bias type is reduced [71].

In conclusion, we observed increased risk of cryptorchidism among boys after maternal airborne LC-PCB exposure. Our results indicate LC-PCB might induce developmental toxicity, but due to the proxy nature of our measure of exposure, inability to perform dose–response analyses, and the lack of comparable literature, larger cohort studies are needed to investigate the safety of staying in PCB contaminated residences and workplaces up to and during pregnancy.

## Data Availability

Data was available from the HESPAIR cohort established in 2019 by the Department of Occupational and Environmental Medicine (Bispebjerg Hospital, Denmark) and Danish health registers provided by Statistics Denmark.
